# Deterministic Localization for Fully Automatic Operation: A Survey and Experiments

**DOI:** 10.3390/s24134128

**Published:** 2024-06-25

**Authors:** Wan-Ning He, Xin-Lin Huang

**Affiliations:** 1 Department of Information and Communication Engineering, Tongji University, Shanghai 200092, China; 2111103@tongji.edu.cn; 2 Shanghai Institute of Intelligent Science and Technology, Tongji University, Shanghai 200092, China

**Keywords:** deterministic localization, certainty, interference mitigation, anchor deployment

## Abstract

With the rapid development of fully automatic operation (FAO) and location-based services, the evaluation criteria of average localization accuracy can no longer meet our demands, in favor of deterministic localization. However, most localization researches modeled localization performance function and enhanced it by minimizing average localization root mean square error (RMSE). The performance degradation in a small region was not considered. In this paper, we present a survey of deterministic localization and analyze the relationship between accuracy and certainty. In this paper, two common solutions of localization enhancement are presented and their localization certainties are discussed. Furthermore, we carry out related localization enhancement experiments in rail transit line and analyze their improvement on deterministic localization. The experimental results show that the overall localization performance is improved, while the deterministic localization requires the stricter solution to promote.

## 1. Introduction

Currently, the Intelligent Transport System (ITS) heavily relies on location information, which is the key to safety [1,2]. The global navigation satellite system (GNSS) is the most common technology in the transport field. However, it has some challenges in terms of real-time, accuracy, reliability and safety, especially in some harsh scenarios (e.g., tunnel, underground and urban canyon) [3]. Besides, with the popularization of fully automatic operation (FAO), high accuracy and deterministic localization are all-important (e.g., 95% localization error within 0.48 m on freeways and higher accuracy demands on local street) [4]. To address these issues, multiple localization methods have been gradually emerging as the complement of GNSS, such as wireless localization [5,6], visual localization [7], inertial navigation system (INS) [8] and so on. Alternatively, some researches utilize sensor fusion, map-matching and other methods to achieve localization enhancement [9].

Wireless localization technology measures ranging or angles and calculates the target’s position by radio wave [10], which mainly includes long-term evolution (LTE) [11], ultra-wide band (UWB) [12], Wi-Fi [13] and Bluetooth [14]. Compared with other techniques, it relies less on the environment and has been thought of as a promising complement of GNSS. However, during the wireless propagation and position solution, the anchor deployment and non-line-of-sight (NLoS) propagations have a significant impact on its localization performance [15,16]. As a result, some relevant researches aim to minimize the average localization root mean square error (RMSE) by anchor deployment optimization [17,18] or NLoS interference mitigation [19,20].

In fact, Level 2+ autonomous vehicles require a lateral error bound of 1.11 m (0.44 m, 95%) for lane determination and 0.41 m (0.16 m, 95%) for lane keeping [21]. On local streets, the road geometry makes requirements stricter, with a lateral error bound of 0.89 m (0.36 m, 95%) for lane determination and 0.21 m (0.08 m, 95%) for lane keeping [21]. As a result, the localization enhancement based on the average error (i.e., MSE or RMSE) is no longer sufficient for the autonomous vehicle. The error bound and certainty should be further taken into account. In [22], an anchor deployment solution was proposed, which aimed at minimizing the localization error bound under a given sensor number. Reference [23] proposed a D-optimality design and minimized the uncertainty ellipsoid. During the optimization based on the error bound and the uncertainty function, the localization certainty and safety could be promoted. In mixed LoS/NLoS scenarios, the corresponding cumulative distribution functions (CDF), before and after being handled by the NLoS detection and mitigation algorithm, were further analyzed [24,25]. The experiments indicated that the NLoS mitigation algorithm decreased the uncertainty of localization to some degree and improved the localization stability.

Regarding localization for GNSS-deprived zones, where the GNSS signal is occluded or even cannot be received, there are a number of relevant surveys, including [26,27,28,29,30,31,32,33,34]. Most focus on localization techniques and their accuracies. Some overviews of improved localization solutions were presented, respectively, with wireless network [26], UWB [27] and radio frequency identification (RFID) technologies [30]. On the basis of academic researches, ref. [28] considered the use case and comprehensively analyzed the commercial trends of wireless localization. Ref. [29] analyzed the fundamental limitations, including theoretical and practical limitations, and listed their recent solutions. They were greatly significant for the practical application. Regarding the solution of localization enhancement, the mixed LoS/NLoS scenarios were taken into account in [31], where the interference mitigation solutions were reviewed. With the prior knowledge of existing maps, an overview of the map matching localization algorithm was provided [32], which was thought as one of the most accuracy solutions for self-driving. Besides, the V2X-based cooperative localization methods were discussed in [34], and these methods could reach high accuracy with low computation complexity. In particular, ref. [33] classified indoor localization schemes into crowdsensing and crowdsourcing categories, which discussed the levels of user contribution and device intervention.

From the current existing surveys, the localization accuracy of different solutions has been widely discussed and compared. However, there is a lack of reviews on deterministic localization. In deterministic localization, we evaluate its performance by not only the average localization error but also its confidence interval and error bound. In the autonomous driving field, there are certain hidden dangers in the evaluation standards of the average accuracy. The sharp localization degradation in a small region is adverse to its safety. As a result, the confidence interval and certainty in deterministic localization are extremely significant. In this survey, the objective is to provide a comprehensive overview of localization for the GNSS-deprived zone, mainly including localization techniques, enhancement solutions and highlighting the localization certainty analysis. The key contributions include the following:

(1) We provide an overview of the localization techniques and solutions of accuracy enhancement. The localization certainty of common solutions is discussed, mainly including anchor deployment optimization and NLoS interference mitigation. We point out that deterministic localization requires the stricter solution to promote, but it cannot be ignored in the future.
(2) The related localization enhancement experiments are carried out on the rail transit line. As a result, deterministic localization is analyzed from both academic researches and well-established solutions in practice.

The rest of this survey is structured as follows. In Section 2, we take an overview of the localization fundamentals, including commercial technologies and position-solving algorithms. In Section 3, the anchor deployment optimization problem is presented. Its improvements in accuracy and certainty are analyzed. In mixed LoS/NLoS scenarios, the common interference mitigation algorithms are listed in Section 4. Furthermore, we choose the UWB localization technology as an example. Based on solutions in Section 2, Section 3 and Section 4, we carry out localization enhancement experiments in practice and illustrate the localization certainty in Section 5. Finally, we conclude this survey in Section 6.

## 2. Wireless Localization Fundamentals

The wireless localization mainly includes basic localization technologies, such as LTE, UWB, Bluetooth, Wi-Fi and their hybrid methods. The corresponding position-solving algorithms contain time of arrival (ToA), time difference of arrival (TDoA), angle of arrival (AoA) and received signal strength (RSS). In this section, we outline the main localization technologies, which use the known location or orientation of anchors. Then, we discuss measurements from radio signals in wireless network and present a brief discussion of localization techniques. Finally, the certainty of localization is analyzed.

### 2.1. Basic Localization Technologies

In previous researches and commercial solutions, the localization schemes and the corresponding accuracies are listed in Table 1.

(1) *LTE localization*: By 2025, LTE is expected to handle 4.4 billion users and also remain as the primary role of cellular technology [35]. In addition to communication applications, the flight time and strength of LTE signals can be measured for mobile target localization [36]. With multiple measured time and strengths from different LTE nodes, ToA/TDoA and fingerprint algorithms were utilized to solve the target’s position, which could be enhanced by deep-learning or machine-learning frameworks [35,37]. Among them, ref. [37] proposed an outlier detection and correction algorithm, which exploited trajectory context. Compared with other localization technologies shown in Table 1, it has a lower cost and better coverage in both indoor and outdoor scenarios. However, its localization accuracies are poor and trajectories fluctuate violently, especially in large-scale areas [38,39].

(2) *UWB localization*: UWB has emerged as a promising localization technology and can offer decimeter-level ranging [40]. With the ranging results, ToA and TDoA techniques were widely used to estimate the target’s location [41]. Compared with other localization technologies, it has certain advantages in terms of accuracy in both outdoor and indoor scenarios. However, NLoS propagation has a significant impact on its localization accuracy, and localization in circumscribed regions, such as narrow spaces, is particularly challenging [19,42]. Regarding localization with NLoS propagations, most detected interferences and discarded outliers, where ranging results were not fully utilized. In [43], a Schmidt KF was used to compensate the bias caused by NLoS propagation, instead of discarding. Considering mixed LoS/NLoS scenarios, a joint LoS/NLoS interference detection and mitigation algorithm was proposed in [19], which reduced the dynamic interference to some extent. Besides, the deployment strategies of UWB anchors are also particularly important to the localization performance and robustness. Geometric dilution of precision (GDOP) was introduced in [12] and the localization performance was enhanced by minimizing it.

(3) *Bluetooth localization*: According to the statistics from the Bluetooth Special Interest Group, there will be no less than 7.6 billion Bluetooth devices by 2027 [44]. Bluetooth devices have been widely applied in electronic devices, such as smart phones, laptops, wristbands and so on. Most commercial solutions calculated the target’s location with measured angles and signal strengths [45,46]. Among them, the Spotlight algorithm was proposed [47], which utilized both the AoA and elevation of angle (EoA) of the signals to solve more accurate angle estimates. Considering the ground-reflection, an RSSI-based localization technique was formulated as an optimization problem in [48]. Then, a fingerprint feature extraction (FPFE) was proposed in [49], which applied either the autoencoder (AE) or principal component analysis (PCA). Besides, ref. [50] further introduced the confidence interval for the fingerprint method instead of the average localization error model. Its localization certainty was significantly improved. Among these localization technologies, Bluetooth-based localization outperforms with low power-consumption, low equipment cost and easy deployment [47]. However, it is designed for short-range wireless communication and has challenges in large outdoor areas.

(4) *Wi-Fi localization*: With the increasing demand for Internet access, Wi-Fi has been ubiquitous and pervasive. It can be regarded as a promising localization solution [51,52]. Most existing Wi-Fi localization methods have focused on the RSS-based fingerprint method, which was sensitive to the changing environment [53]. To address this issue, ref. [53] fused on the fingerprint of RSS with multiple classifiers and constructed a two-layer fusion profile. A WiFi-based group of fingerprints (GOOFs) was further constructed to estimate the target’s location in [54], which consisted of RSS, signal strength difference and hyperbolic location fingerprint. In particular, some researches exploited the channel state information (CSI) of the Wi-Fi signal and obtained the relationship between CSI characteristics and accurate locations [55]. Ref. [56] used only one anchor point and proposed three quick remedies to reduce the effects of a dynamic environment. Regarding location tracking, ref. [57] replaced the commonly used filter methods with factor graph optimization. It realized the decimeter-level localization within a small area. As we can see from Table 1, Wi-Fi-based localization technology is mainly applied in indoor scenarios and difficult to achieve large-scale outdoor coverage.

(5) *Mixed localization*: Considering the limitations of the single sensor, multiple sensors are integrated for high accuracy, robustness and complementarity. In addition to the above wireless localization technologies, the inertial measurement unit (IMU) is widely used with low cost and high accuracy. However, with the cumulative error, its localization precision cannot be maintained for a long time [58]. In order to address this issue, some solutions incorporated the wireless location methods with IMU [59,60]. Among them, ref. [61] combined the deep neural network to stabilize IMU data, which overcame its cumulative errors. Then, the impact of NLoS propagation on fusion performance was further taken into account in [62], where the number of anchors was adjusted adaptively by discarding ranging outliers. Besides, ref. [25] combined Wi-Fi fingerprint-based localization with UWB range-based localization and effectively mitigated the NLoS interferences. From Table 1, we can see that the mixed localization schemes can realize mutual complementary advantages and achieve better performance under interferences.

**Table 1:** Solutions for wireless localization.

Localization Technology	Scheme	Region	Accuracy	Reference
LTE	Deep-learning-based framework	6.27 km^2^, outdoor	13.18 m	[35]
	Fingerprint of RSS and KF	30 m × 40 m, indoor	2.78 m	[36]
	TDoA and fingerprint of RSS	30 m × 10 m, indoor	≈1.00 m	[11]
	Fingerprint of CSI and deep learning	3.6 m × 6 m, indoor/360 m × 195 m, outdoor	0.47 m/19.90 m	[39]
	Machine learning and outlier correction	outdoor	32.2 m	[37]
	TDoA, JMM and KF	700 m × 800 m, outdoor	19.00 m (@67%) *	[38]
	ToA and probabilistic machine-learning	120 m × 80 m, outdoor	≤10.00 m	[63]
UWB	ToA and derivative UKF	10 m × 10 m, indoor	0.05 m	[42]
	TDoA and clock drift elimination	128 m^3^, indoor	0.08 m	[40]
	Bias compensation and cooperative localization	60 m × 60 m, outdoor	0.87% failure	[43]
	MLE and anchor deployment optimization	1.2 m × 3.2 m × 2.6 m, indoor	0.15 m	[12]
	ToA and LoS/NLoS interference mitigation	30 m × 5 m, outdoor	0.38 m	[19]
	Deep location network and ranging correction	6 m × 5 m, indoor	0.23 m	[64]
	TDoA and semidefinite relaxation	14 m × 13 m	2.20–2.50 m	[41]
	RSS and enhanced geometric filtering	6.6 m × 5.4 m, indoor	0.16 m	[65]
Bluetooth	AoA and self-localization	12 m × 12 m, outdoor	3.60 m	[45]
	FPFE based on Minkowski distance	5 m × 8 m, indoor	0.68 m	[49]
	RSS measurements and multilateration	6.15 m × 28.15 m, indoor	3.80 m	[46]
	AoA and elevation-of-angle	3 m × 10 m × 3 m, indoor 10 m × 13 m × 3 m, indoor 20 m × 25 m × 8 m, indoor	0.48 m 0.67 m ≈0.48 m	[47]
	Confidence-interval fuzzy model	85 m^2^, indoor	1.00 m (@97%)	[50]
	RSS-based and optimization	100 m × 100 m, outdoor	0.78–1.68 m	[48]
Wi-Fi	Wi-Fi fingerprint-based and KNN	≈1000 m^2^, indoor	3.11 m	[51]
	Feature fusion by channel state information	30 m^2^/10 m × 2 m, indoor	0.8 m/1.1 m	[52]
	Fingerprint of RSS and multiple classifiers	73 m × 20 m, indoor	2.50 m	[53]
	Group of fingerprints, including RSS	308.4 m^2^, indoor	2.79 m	[54]
	AoA and co-localization algorithm	16 m × 10 m/8.5 m × 5.5 m/ 10 m × 8 m/6 m × 2.1 m, indoor	0.35 (@50%)	[66]
	Fusion of multiple channel state information	6 m × 7.8 m/3.5 m × 4.2 m, indoor	1.62 m/2.41 m	[55]
	Round-trip phase and factor graph optimization	4 m × 4 m	0.26–0.56 m	[57]
	Channel frequency response database and fingerprint	1 m × 1 m	0.05 m	[56]
Mixed	RSS of LTE, odometry and particle filter	500 m × 500 m, outdoor	13.07 m	[67]
	Tightly coupled visual–inertial–UWB	11 m × 11 m, indoor	0.21–0.66 m	[62]
	UWB ranging and Wi-Fi fingerprint	30 m × 30 m, indoor	≤0.85 m	[25]
	UWB ranging, IMU and tight integration	120 m × 200 m, outdoor	2.55 m	[59]
	Data-driven IMU and Bluetooth	52.5 m × 52.5 m, indoor	1.01–1.46 m	[61]
	Wi-Fi, Bluetooth and HTF algorithm	62 m × 28 m, indoor	1.25–2.29 m	[68]
	Wi-Fi, IMU and LSTM algorithm	10 m × 15 m, indoor 50 m × 2 m, indoor	0.51–1.17 m 1.35–2.16 m	[60]

* i.e., “19.00 m @67%” means that 67% localization errors are within 19.00 m.

### 2.2. Basic Localization Techniques

**Figure 1:**
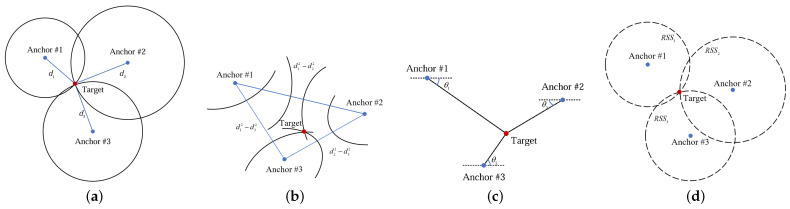
The basic localization techniques: (**a**) ToA technique. (**b**) TDoA technique. (**c**) AoA technique. (**d**) RSS technique.

In this part, we outline the main range-based techniques for wireless localization and discuss some range-free localization methods. With the given location and the antenna orientation of anchor nodes, we briefly discuss the basic localization techniques, illustrated in Figure 1, including ToA, TDoA, AoA and RSS.

(1) *ToA technique*: According to the communication between anchor nodes and the target, the transmission time $t_{i}$ of the signal between the *i*th (i.e., Anchor #i in Figure 1) anchor and the tag can be measured. The ranging results can be calculated with their transmission time multiplied by the speed of electromagnetic wave *c*, expressed as

(1) $$d_{i}=c\times t_{i}.$$

With the given locations of reference anchor nodes, each ranging result provides a circle with the reference anchor node as its center. In the Cartesian coordinate system, we assume that there are *N* anchors. Let the reference anchor be $a_{i}={\left(x_{i},y_{i}\right)}^{T}$, i=1,2,...,N and the tag be $x={\left(x,y\right)}^{T}$. The circle in Figure 1a can be represented as

(2) $${\left(x_{i}-x\right)}^{2}+{\left(y_{i}-y\right)}^{2}=d_{i}^{2}.$$

With the first anchor as a reference, take Equation (2) minus $d_{1}^{2}$ for each *i* and construct the ToA equation as

(3) $$\begin{array}{c} Ax=2\left(\begin{array}{cc} x_{2}-x_{1} & y_{2}-y_{1}\\ x_{3}-x_{1} & y_{3}-y_{1}\\ \vdots & \vdots \\ x_{N}-x_{1} & y_{N}-y_{1} \end{array}\right) \end{array}x=\begin{array}{c} \left(\begin{array}{c} d_{2}^{2}-d_{1}^{2}-∥a_{2}{∥}_{2}^{2}+{∥a_{1}∥}_{2}^{2}\\ d_{3}^{2}-d_{1}^{2}-∥a_{3}{∥}_{2}^{2}+{∥a_{1}∥}_{2}^{2}\\ \vdots \\ d_{N}^{2}-d_{1}^{2}-∥a_{N}{∥}_{2}^{2}+{∥a_{1}∥}_{2}^{2} \end{array}\right)=b \end{array}.$$

Here, A and b are, respectively, the observation matrix and the measurement vector. As a result, the intersection of multiple circles is the target’s location, which can be solved by the least square method, $x=A^{†}b$. Here, $A^{†}$ is the pseudo inverse matrix of matrix A, i.e., $A^{†}={\left(A^{T}A\right)}^{-1}A^{T}$.

(2) *TDoA technique*: Similar to the ToA technique, the TDoA technique also calculates ranging results by the transmission time. However, it uses the time difference between two reference anchor nodes and the target is on the hyperbolic curve with their ranging difference, expressed as

(4) $$d_{i}-d_{1}=\sqrt{{\left(x_{i}-x\right)}^{2}+{\left(y_{i}-y\right)}^{2}}-\sqrt{{\left(x_{1}-x\right)}^{2}+{\left(y_{1}-y\right)}^{2}}.$$

Therefore, the intersection of multiple hyperbolic curves can be solved as the coordinate of the target. The common method is the Chan method [69].

(3) *AoA technique*: Different from other techniques, it is an angle-based method. With the antenna array equipped on nodes and *N* anchors, the orientation angle can be observed, expressed as $\theta_{i}$, i=1,2,...,N. The directional line of the target can be obtained, shown in Figure 1c, and satisfies

(5) $$\tan\theta_{i}=\frac{y-y_{i}}{x-x_{i}}.$$

Then, with multiple measured angles and corresponding directional lines, the AoA equation can be constructed as

(6) $$\begin{array}{c} 2\left(\begin{array}{cc} -\tan\alpha_{1} & 1\\ -\tan\alpha_{2} & 1\\ \vdots & \vdots \\ -\tan\alpha_{N} & 1 \end{array}\right) \end{array}x=\begin{array}{c} \left(\begin{array}{c} y_{1}-\tan\alpha_{1}x_{1}\\ y_{2}-\tan\alpha_{2}x_{2}\\ \vdots \\ y_{N}-\tan\alpha_{N}x_{N} \end{array}\right) \end{array}.$$

Similarly, the coordinate of the target can also be solved by least square method.

(4) *RSS technique*: When the transmission power is fixed, we construct the relationship between the distance and the received power, expressed as

(7) $$RSS_{i}=RSS_{d_{0}}-10n\mathrm{lg}\left(\frac{d_{i}}{d_{0}}\right)=C-10n\mathrm{lg}\left(d_{i}\right).$$

Here, $RSS_{d_{0}}$ represents the reference signal strength with transmission distance $d_{0}$ and the variable *C* is a constant. The ranging results between anchor nodes and the target can be estimated by signal strengths $RSS_{i}$. The target’s location can be solved in the same way as the ToA technique.

Unlike the above technique, range-free localization techniques utilize the reference anchor’s location and detect the tag with the presence or absence identification. According to the correlation information between the anchors and the target, it can make a rough estimate of the tag’s location. These methods can be implemented simply at low cost under the condition of low positioning accuracy, such as centroid [70], APIT [71], DV-hop [72] and so on.

Due to the occasional blocking, NLoS propagation is sometimes experienced. As a result, the corresponding ranging results, angle results and received strengths fluctuate dramatically. The circles, hyperbolic curves and directional lines in Figure 1 cannot intersect at one point. Therefore, some researches focus on the high-accuracy localization under NLoS interference or modify the anchor node deployment to enhance localization robustness.

## 3. Localization Enhancement by Anchor Deployment Optimization

During the wireless localization, the node deployment of the wireless network has a significant impact on the localization performance and robustness. Viewed from the current solutions of anchor deployment optimization, they can be classified into two types: (1) constructing the localization performance function as the optimization objective and optimizing anchor deployment directly; (2) selecting the optimal anchor group from multiple candidate locations. The related solutions in recent researches are listed in Table 2.

In Type 1, most researches constructed the localization performance function based on the average Cramer–Rao lower bound (CRLB) [73,76], which could promote the improvement of the general localization performance. In complex scenarios with fixed obstacles, a robust 3D localization method was proposed in [77], which could not only accommodate the outliers but also optimize the anchor deployment. Besides, ref. [74] further constructed a Deep Q-Learning Energy-optimized LoS/NLoS (DQLEL) framework to solve the complex optimization problem. It achieved a balance of localization performance and battery life in mixed LoS/NLoS scenarios. This optimization method can directly obtain the global optimal solution, but it is challenging for it to solve optimization problems with multiple objectives in complex scenarios. This type of solution can be applied in large open areas without fixed obstacles or obstruction.

In Type 2, the optimal anchor groups were always selected from the preset grid candidate positions [80,81]. Among them, ref. [79] considered the harsh indoor scenario with the corner and solved the optimal deployment strategy by genetic algorithm. Besides, the weight position dilution of precision was studied in [17] and, based on it, the optimal deployment scheme was chosen from different projection shapes of deployed anchors. The solutions of this method may not be the global optimal, but complex optimization problems (e.g., mixed integer optimization problem) can be solved easily. These solutions achieve better performance in indoor rooms, corners or other harsh scenarios, with some fixed obstacles.

View from the current researches, the average localization error is always set as the optimization objective. It will ignore localization failure within a small area and is adverse to deterministic localization. In order to enhance the localization certainty, it needs to be strengthened on optimizing anchor deployment based on localization confidence intervals. During the deterministic localization, the optimization objective should be modified as the upper bound of the confidence interval instead of the average localization error along the trajectory. However, with the anchor-group switch on a large scale, it is challenging to find the certain location corresponding to the upper bound. The optimization problem will be more complex to solve with higher computation complexity.

## 4. Localization Enhancement by LoS/NLoS Interference Mitigation

Further considering NLoS propagations and measurement noise, the LoS/NLoS interference mitigation is especially significant for localization enhancement. From the related researches, the mitigation solutions can be also summarized into two types: (1) eliminating the NLoS impact on localization directly without any interference identification; (2) identifying the interference firstly (i.e., LoS/NLoS detection) and discarding or eliminating the outliers. The corresponding solutions are listed in Table 3.

In Type 1, the typical Chan localization algorithm was proposed in [69] and followed by a variety of improved schemes [83,84]. They are more available for strong interference environments. Besides, some localization optimization problems were constructed with the constraints of NLoS bias and relaxed them into a convex optimization [85,87]. Among them, total least square, instead of the commonly used least square method, was utilized as the initial of programming in [86]. It improved localization accuracy and convergence speed. These methods can eliminate not only noise measurement but also NLoS bias. However, they cannot detect outliers from localization results, which will have a negative effect on fusion or some other subsequent operations. This type of solutions can be more accurate and can be applied as the final output without the following processing.

In Type 2, regarding the LoS/NLoS detection, some machine learning methods were used for high-accuracy identification [20,92]. Among them, the ranging error was calculated by the assumption of single NLoS propagation and iteratively updated for multiple NLoS propagations in [90]. The LoS/NLoS interference could be identified by a proper threshold. In particular, assuming the NLoS as the sparse outlier, ref. [89] introduced the sparse optimization for detection and mitigation. Regarding the interference mitigation, some researches selected the anchor group based on identified LoS/NLoS results and increased the utilization of LoS propagations [91]. These methods can realize the channel classification, which is beneficial for subsequent processing. However, the identification error will also introduce interferences for localization. They are superior for subsequent processing or integration from multiple localization results, such as fusion with other sensors, map matching and so on.

Based on the current researches, the NLoS bias can always be eliminated effectively and the localization error will decrease. In addition, the further analysis of localization certainties provides great significance for fusion localization or subsequent processing. It can be set as another evaluation criteria. However, most recent works reduced the interference by nonlinear variation, where the relationship of the localization certainties before and after handling could be difficult to express. Hence, the confidence interval of localization error after handling by the interference mitigation solution is more challenging to calculate numerically.

## 5. Experimental Evaluation of UWB Localization

In this section, UWB localization is selected as an example and its localization experiments on the Zhangjiang Rail Transit Test Line are evaluated. In the experiments, we use the ToA localization technique in Section 2 and further enhance localization by the two mentioned types of solutions in Section 3 and Section 4 (i.e., optimizing anchor deployment and interference mitigation). The root mean square error (RMSE) is set as the evaluation criteria, which is defined as

(8) $$RMSE=\sqrt{\frac{1}{M}\sum_{i=1}^{M}{\left({\hat{x}}_{i}-x_{i}\right)}^{T}\left({\hat{x}}_{i}-x_{i}\right)}.$$

**Figure 2:**
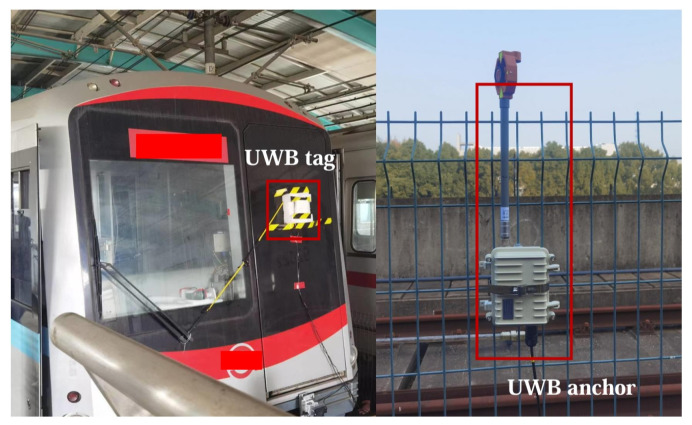
The experimental environment and UWB equipment.

Here, *M* is the sampling times of experiments in the same point. The vector $x_{i}$ and ${\hat{x}}_{i}$ are practical and solved locations. The experimental environment and the equipment deployment are shown in Figure 2. We have deployed raster along the track to capture the relationship between the practical and calculated locations. All UWB anchors and rasters are connected through a switch to realize the time synchronization.

In experiments, we firstly optimize the UWB anchor deployment to enhance its localization, especially in the station region. Secondly, the strong metal interference is modeled and mitigated. Finally, we analyze its deterministic localization enhancement with the above methods and discuss its practicality.

### 5.1. Anchor Deployment Optimization with ROI Enhancement

We assume the UWB anchors are deployed along the track and some anchors behind the train can be occluded. As a result, the ranging results of UWB anchors in front of the train can only be utilized to solve the train’s location. From our previous work [19], we can calculate the localization error at a given position x, expressed as

(9) $$MSE\left(A,x\right)={\left(A\varphi \right)}^{T}A\varphi$$

where the vector φ can be solved in [19]. The localization MSE is related to the tag’s location and anchor deployment, which can determine the matrix A. With the given trajectory of the track *L*, we can integrate the MSEs along the track and the localization performance can be written as

(10) $$f\left(A\right)=\int_{L}MSE\left(A,x\right)dl.$$

Considering the diverse demands on the localization accuracy in the station, we introduce a scaling factor w>1 to enhance the impact of station region. The localization performance function can be modified as

(11) $$f\left(A\right)=w\int_{L_{station}}MSE\left(A,x\right)dl_{1}+\int_{L_{station}^{-}}MSE\left(A,x\right)dl_{2}.$$

Due to the non-convex optimization objective, we choose PSO to find the optimal anchor deployment scheme, which achieves the minimal value of localization performance function in Equation (11).

**Figure 3:**
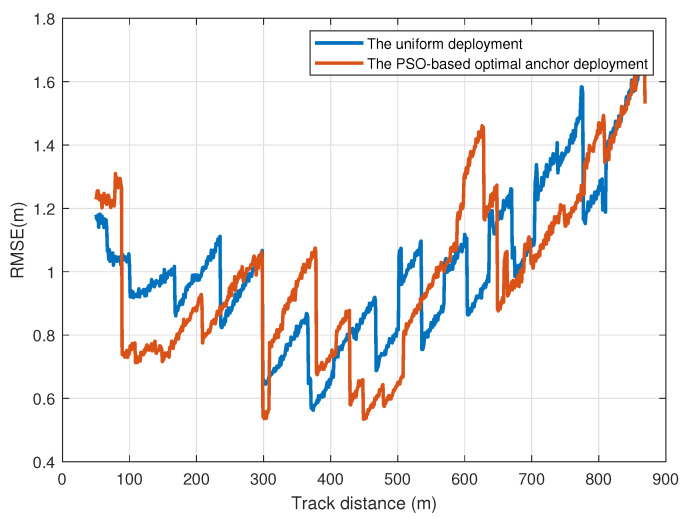
The localization performances of uniform and PSO-based optimal deployment.

The experimental region is 870 m × 4 m, consisting of a 323 m straight track and a 547 m curved track. Among them, the station region is within 100–250 m. Generally, as the number of anchors increases, the localization performance improves and its enhancement becomes less significant with more than 26 anchors. Comprehensively considering the performance and cost, we deploy 26 anchors in this region, and the communication coverage range of UWB anchors is about 300 m. With the PSO-based optimal anchor deployment, the localization performance in the station region is improved. Compared with uniform deployment, the practical localization performances are shown in Figure 3.

As one can see, compared with the uniform deployment scheme on the entire route, the proposed PSO-based optimal deployment achieves 0.02 m gain in terms of RMSE. In particular, within the station region, the proposed deployment achieves 0.16 m gain in terms of RMSE. The localization performance within the station region is improved at the cost of performance degradation in other regions, especially 550–650 m. However, the sudden increase in localization errors has a negative impact on the safety of the system. Hence, the anchor deployment should aim at the deterministic localization, which will ensure the overall availability of the system.

### 5.2. Metal Interference Mitigation and Trajectory Constraint

Since UWB anchors and the tag are mounted on the metal mesh and the train, respectively, there are strong metal interferences during localization. As presented in our previous work [19], we firstly constructed the model of the interference model. Secondly, the ranging error of each propagation path was iteratively updated and corrected. Finally, considering the given track of the train, we introduced a virtual anchor solution. In order to restrict trajectory, we use the anchor with ranging results of 10–30 m to construct the virtual symmetrical anchor. We compare ToA localization techniques with the proposed interference mitigation and the whole scheme (i.e., integrating interference mitigation with virtual anchor). The corresponding localization performance comparisons are shown in Table 4.

As we can see from Table 4, compared with the ToA localization techniques, the localization performance of the proposed interference mitigation algorithm achieves 0.63 m gains in terms of RMSE. After integrating with the virtual anchor solution, the localization error in the vertical track direction is effectively eliminated. The localization performance is further improved by 0.44 m in terms of RMSE.

**Figure 4:**
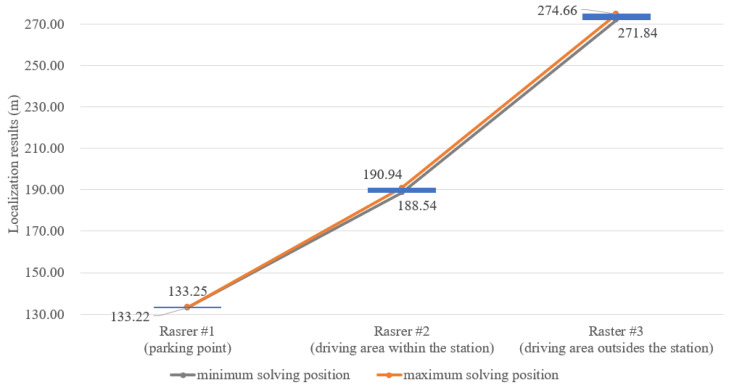
The localization performance of the localization enhancement solution along the track.

Besides, considering the stronger metal interference inside the station, the practical localization performance after being handled by the localization enhancement solution is shown in Figure 4. As we can see from the localization results, the localization performance at the parking point is improved significantly. The interferences are effectively mitigated with relatively dense anchor deployment. However, with sparse deployment of anchor in the other regions, especially the driving area outside the station, the less redundant ranging information is provided and the interferences cannot be totally eliminated. Overall, in order to guarantee the localization performance throughout the entire driving process, the deterministic localization should be more focused on, instead of localization errors.

From the above experimental results, the localization performance can be improve effectively, especially at the parking point. However, the performance degradation in a small region cannot be overcome, which will have a negative impact on the driving availability and safety. Therefore, we need to focus on the stricter solution, i.e., deterministic localization, to promote the localization performance.

## 6. Conclusions

In this paper, we presented a detailed survey on the most current and relevant localization enhancement solutions. The demands on deterministic localization for FAO were analyzed. Firstly, we outlined the localization fundamental, which consisted of commercial localization technologies and basic localization techniques. Secondly, the anchor deployment optimization problem was presented. The two discussed types of solving methods used average localization errors to evaluate their performance but ignored the localization certainty in a small region. Thirdly, in mixed LoS/NLoS scenarios, the current interference mitigation algorithms were listed, which are also classified into two types. They could partly improve the localization certainty but could not guarantee the deterministic localization. Finally, with UWB localization as an example, we carried out localization enhancement experiments in practice and illustrated that the deterministic localization requires the stricter solution to maintain and promote. As a result, the accuracy-focused localization will develop towards deterministic localization in the future.

## 7. Future Work

In the future, we will focus on joint optimization of the number of anchors and the anchor deployment based on deterministic localization. Besides, we will further provide a survey that outlines the key findings and recommendations for deterministic localization enhancement.

## Figures and Tables

**Table 2:** Solutions for anchor deployment optimization.

Type	Scheme	Application Scope	Reference
Type 1	CRLB as localization performance function and using gradient descent method to solve optimal solution	ToA	[73]
	Multi-objective optimization for anchor deployment, power allocation and solving by BPSO	ToA and AoA	[22]
	Considering LoS/NLoS propagations and balancing localization error and battery life	TDoA	[74]
	Joint optimization for minimizing localization time and region coverage	ToA	[75]
	Constructing the relationship between distance and noise, and minimizing average CRLB	ToA	[76]
	In the present of outliers, optimizing the combination of Fisher information matrices	RSS	[77]
	Converting the anchor deployment optimization into finding an ensemble of bipartite graphs	RSS	[78]
Type 2	Presenting weight position dilution of precision and evaluating the best projection shapes of deployed anchors	ToA	[17]
	Selecting the optimal anchor group by genetic algorithm in a complex scenario with a corner	ToA and AoA	[79]
	Predefined region of interest for obstacles, and joint optimization for NLoS effects and the number of anchors	TDoA	[80]
	Joint optimization for localization performance, unique fingerprint and minimum number of anchors	RSS	[81]
	With fixed number of anchors, selecting optimal anchor deployment by minimizing localization error uncertainty	ToA	[82]
	Analysis of anchor deployment with geometric dilution of precision, Fisher information matrix and RMSE	ToA	[12]

**Table 3:** Solutions for LoS/NLoS interference mitigation.

Type	Scheme	Application Scope	Reference
Type 1	Chan algorithm and its improvements by particle swarm optimization	TDoA	[69,83,84]
	Constructing a weighted least squares problem and relaxing it into a convex semidefinite program	ToA	[85]
	Total least square integrated with a regularization term and converted into a semidefinite program	ToA	[86]
	Formulating a robust least square and the convex relaxation-based approximation method	TDoA	[41]
	Considering the anchor location errors and convex relaxations for formulated maximum likelihood problem	TDoA	[87]
	Developing a semidefinite program without prior information of LoS/NLoS channel	ToA/TDoA	[88]
Type 2	Sparse optimization with L1-norm minimization and solving it by alternating direction method of multipliers	ToA	[89]
	LoS/NLoS detection by fuzzy comprehensive evaluation and equality estimating location by ECTSRLS algorithm	ToA	[20]
	Markov decision process and select the anchor nodes with LoS propagation according to deep Q-learning	TDoA	[74]
	Iterative algorithm for ranging error estimation and LoS/NLoS identification with the error tetrahedron	ToA	[90]
	Distributed filtering for NLoS identification and hybrid particle/finite impulse response filter for failure	ToA	[91]
	Variational autoencoder for NLoS detection and EKF based on the predicted score	TDoA	[92]

**Table 4:** Localization performance comparisons.

Localization Scheme	Location 1 (cm)	Location 2 (cm)	Location 3 (cm)	Location 4 (cm)	Average RMSE (cm)
Practical location	(200, 1058)	(200, 1160)	(200, 1817)	(200, 1908)	/
ToA technique	(271.7, 1102.9)	(258.2, 1199)	(−11.6, 1914.9)	(97.3, 1995.4)	145.4
Interference mitigation	(306.3, 1090.8)	(291.6, 1184)	(149.5, 1861.3)	(196, 1944.5)	82.5
Whole scheme	(208.9, 1101.4)	(207.6, 1193.2)	(195.8, 1856.3)	(199.7, 1944.1)	38.7

## Data Availability

The figures and localization data were sourced from at https://github.com/HeWanning/Deterministic-Localization-for-Fully-Automatic-Operation-A-Survey-and-Experiments.git (accessed on 21 June 2024).
